# CAR-NK Engineering to Overcome TME Barriers

**DOI:** 10.3390/cells15010021

**Published:** 2025-12-22

**Authors:** Fahmida Islam, Aleta Pupovac, Richard L. Boyd, Alan O. Trounson

**Affiliations:** 1 Cartherics Pty Ltd., Notting Hill, VIC 3168, Australia; fahmida.islam@monash.edu (F.I.); aleta.pupovac@monash.edu (A.P.); alan.trounson@monash.edu (A.O.T.); 2 Australian Regenerative Medicine Institute, Monash University, Clayton, VIC 3168, Australia

**Keywords:** CAR-NK cells, TME, antigen heterogeneity, immune escape

## Abstract

Chimeric antigen receptor (CAR)-based immunotherapy has shown considerable promise in cancer treatment by redirecting immune effector cells to recognize and eliminate tumor cells in an antigen-specific manner. While CAR-T cells bearing tumor-specific CARs have shown remarkable success in treating some hematological malignancies, their clinical application is limited by cytokine release syndrome, neurotoxicity, and graft-versus-host disease. In contrast, CAR–natural killer (NK) cells retain their multiple forms of natural anti-tumor capabilities without the pathological side effects and are compatible with allogeneic “off-the-shelf” application by not requiring prior activation signaling. Despite CAR-NK therapies showing promising results in hematological malignancies, they remain limited as effector cells against solid tumors. This is primarily due to the complex, immunosuppressive tumor microenvironment (TME), characterized by hypoxia, nutrient depletion, lactate-induced acidosis, and inhibitory soluble factors. Collectively, these significantly impair NK cell functionality. This review examines challenges faced by CAR-NK therapy in combating solid tumors and outlines strategies to reduce them. Barriers include tumor antigen heterogeneity, immune escape, trogocytosis-mediated fratricide, rigid structural and metabolic barriers in the TME, immunosuppressive factors, and defective homing and cell persistence of CAR-NK cells. We also emphasize the impact of combining other complementary immunotherapies (e.g., multi-specific immune engagers and immunomodulatory agents) that further strengthen CAR-NK efficacy. Finally, we highlight critical research gaps in CAR-NK therapy and propose that cutting-edge technologies are required for successful clinical translation in solid tumor treatment.

## 1. Introduction

**Figure 1:**
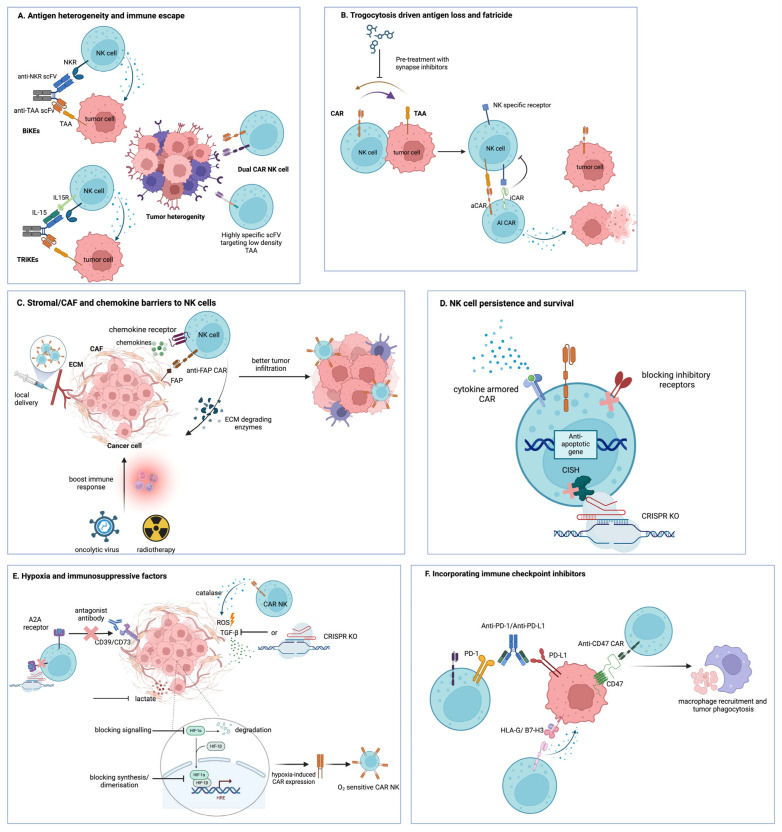
Summary of CAR-NK engineering strategies to overcome major TME barriers in solid tumors. (**A**) Antigen heterogeneity and antigen-low tumor cells are addressed by NK cell engagers (BiKEs and TriKEs), which bridge NK receptors to tumor-associated antigens (TAAs) and, in the case of TriKEs, deliver IL-15 to support NK survival, as well as dual-CAR-NK cells and highly specific scFvs that broaden target recognition of low-density TAAs. (**B**) Trogocytosis-driven antigen loss and CAR-NK fratricide are limited by AI-CARs based on NK-specific inhibitory receptors and by pharmacological inhibition of immune synapse formation. (**C**) Stromal, CAF, and chemokine barriers are targeted using FAP-specific CAR-NK cells, ECM-degrading enzymes, chemokine receptor engineering, oncolytic viruses, radiotherapy, and local delivery strategies to remodel stroma and enhance NK cell infiltration. (**D**) Hypoxia and metabolite-mediated suppression in the TME are countered by hypoxia-inducible CAR-NK designs, antioxidant enzymes, such as catalase, and the blockade of adenosine-generating pathways (CD39/CD73) or A2A receptors. (**E**) CAR-NK persistence and survival are enhanced by CISH knockout and cytokine-armored CARs that provide more sustained cytokine signaling. (**F**) Inhibitory checkpoint pathways are overcome by antibody blockade or CAR-mediated targeting of PD-1/PD-L1, HLA-G, B7-H3, and CD47, promoting tumor cell clearance, including macrophage-mediated phagocytosis. Created with BioRender.com (https://www.biorender.com/).

Chimeric antigen receptor (CAR)–natural killer (NK) cells carry lower risks of cytokine release syndrome, neurotoxicity, and graft-versus-host disease than CAR-T cells and are compatible with allogeneic, off-the-shelf production. However, efficacy in solid tumors remains constrained by the immunosuppressive and physically restrictive tumor microenvironment (TME) [1]. This review discusses the main barriers that limit the effectiveness of CAR-NK cells in the solid TME, including antigen heterogeneity, immune evasion, metabolic suppression, trogocytosis-induced fratricide, and inadequate cell persistence We also outline novel engineering and combinatorial strategies designed to reduce these hurdles and advance CAR-NK therapy towards successful clinical application in solid tumor settings (summarized in Table 1 and Table 2 and Figure 1).

## 2. NK Cells in the TME

The TME represents a complex, highly immunosuppressive environment that leads to significant challenges to the function and therapeutic efficacy of NK cells. The hostile TME comprises various immunosuppressive cells, including regulatory T cells, cancer-associated fibroblasts (CAFs), myeloid-derived suppressor cells (MDSCs), and tumor-associated macrophages (TAMs). Together, these populations limit immune cell infiltration into tumors and dampen their cytotoxic activity through inhibitory cytokines, nutrient depletion, and extracellular matrix (ECM) remodeling [84,85,86,87]. Furthermore, the TME contains numerous inhibitory factors, such as immunosuppressive cytokines, including interleukin (IL)-10 [87], transforming growth factor (TGF)-β [53], metabolic inhibitors, like prostaglandin E2 (PGE2) [88], adenosine [89], and enzymatic suppressors, such as indoleamine 2,3-dioxygenase (IDO), that converts tryptophan into immunosuppressive L-kynurenine metabolites [90,91]. All of these collectively contribute to the immunosuppressive TME, which is further characterized by hypoxia, nutrient depletion, and lactate-induced acidosis. These factors significantly impair NK cell function through several mechanisms, such as downregulation of activation receptors, including NKG2D (natural killer group 2, member D), and the natural cytotoxicity receptors (NKp30, NKp44, and NKp46). They also upregulate inhibitory checkpoints such as CD94/NKG2A, programmed cell death protein 1 (PD-1), T cell immunoglobulin and mucin domain containing-3 (TIM-3), T cell immunoreceptor with Ig and ITIM domains (TIGIT), cytotoxic T lymphocyte-associated protein-4, lymphocyte activation gene 3 (LAG-3), and interleukin 1 receptor 8 [92,93]. In addition, they suppress the production of granzyme B, perforin, and interferon (IFN)-γ [94,95]. They also alter cellular metabolism, inhibiting glycolysis and oxidative phosphorylation [96]. Furthermore, via chemokine expression modulation, the TME promotes the recruitment of less cytotoxic CD56^bright^ NK cells over the more potent CD56^dim^ NK cells, and the stromal cells create a physical barrier that further prevents the infiltration and trafficking of NK cells towards the tumor targets [97]. Therefore, extensive research has been carried out to understand these immunosuppressive mechanisms in order to engineer CAR-NK cells to better equip them to tackle TME-mediated inhibition and enhance immunotherapeutic potential against solid tumors.

## 3. Targeting Antigen Heterogeneity in Solid Tumors and Overcoming Immune Escape

Unlike most hematological malignancies, solid tumors are highly heterogeneous in their antigen expression [98], where they often evade immune surveillance by downregulating their targeted antigens [99]. To reduce these challenges, CAR-NK cells can be designed to either increase the binding affinity of the CAR to the low-expression antigens or co-target multiple tumor-associated antigens (TAAs) (Figure 1A). Various techniques, including directed evolution [2], computational modeling [3,4], and phage display [5,6], have been utilized in CAR-T cell studies to design single-chain variable fragments (scFvs) highly specific to these low-density TAAs, resulting in enhanced efficacy while minimizing off-target toxicities. These platforms can also be leveraged for CAR-NK construct optimization. On the other hand, multi-targeting strategies using two independent “dual” CAR-NK cells have been shown to reduce tumor resistance. Dual CAR-NK cells targeting programmed death-ligand 1 (PD-L1) and human epidermal growth factor receptor (HER2) have shown enhanced cytotoxicity across diverse cancer cell lines, including breast, ovarian, pancreatic, and gastric cancer cells, when compared to mono-specific variants [7]. Similarly, dual CAR-NK cells against CD44-CD24 and CD44–mesothelin illustrated superior killing against triple-negative breast cancer cells, reducing tumor survival to 4–10% compared to 18–25% with single-target CARs [8]. NK cells expressing dual CARs targeting GD2 and NKG2D ligands showed improved recognition of glioblastoma (GBM) cells, achieving a ~2-fold reduction in tumor volume and weight compared to single CAR-treated mice [9]. Similarly, dual-targeting epidermal growth factor receptor (EGFR) and EGFRvIII CAR-NK cells improved the killing of GBM compared to mono-specific CARs [10] (Table 1).

Additionally, multi-specific engagers can be administered along with CAR-NK therapy to address antigenic heterogeneity in solid tumors (Figure 1A). Bispecific killer engager (BiKE) molecules are engineered to simultaneously bind tumor antigens and activate NK receptors such as CD16, NKp30, NKp46, and NKG2D. Building on the BiKEs concept, trispecific killer cell engagers (TriKEs) incorporate an additional functional domain, such as the cytokine IL-15, which further promotes activation, proliferation, and persistence, improving their efficacy in the TME [11]. Various studies have shown the improved cytolytic activity of NKG2D CAR-NK cells when incorporated with BiKEs, achieving over 60% lysis in HER2 breast carcinoma cells [12] and increasing glioblastoma cell killing from 11–13% to 32–43% in EGFR/HER2-targeted models [13]. Current CAR-NK clinical trials show activity against broad/stress-induced targets (for example, PD-L1 and NKG2D) and include multi-antigen recognition strategies (Table 1 and Table 2). In addition to antigen recognition, the CAR construct influences the quality of NK cell function. Incorporating NK cell-specific signaling and adaptor domains, such as 2B4, DNAM-1, DAP10, and DAP12, in combination with NKG2D, enhances activation signaling pathways, promotes degranulation, increases IFN-γ secretion, and improves overall cytotoxicity (Table 1) [14,15].

## 4. Modulating Trogocytosis to Enhance CAR-NK Function

Trogocytosis refers to the transfer of surface proteins, membrane fragments, or cytoplasmic components from another cell via direct cell-to-cell contact without cell fusion or death [100]. Trogocytosis-mediated immune escape has been documented in CAR-NK therapy [17] and occurs in two major ways. Firstly, it involves the transfer of cell surface receptors from cancer targets to the CAR-expressing effector cells, which leads to the downregulation of target antigens on the tumor cells. Secondly, it involves the transfer of CAR molecules to the tumor targets, resulting in antigen masking and depletion of CAR molecules on the engineered immune cells [101]. Furthermore, trogocytosis mediates fratricide, as CAR-bearing cells may target one another due to acquiring the target antigen, thereby reducing cell viability and anti-tumor efficacy.

Trogocytosis of tumor antigens on CAR-bearing cells can upregulate inhibitory molecules, such as LAG-3 and TIM-3, resulting in CAR cell exhaustion and dysfunction [16]. Trogocytosis in CAR-NK cells also causes metabolic dysregulation, leading to a significant suppression of glycolysis [17]. Addressing these challenges, Li and colleagues [17] designed a dual CAR (AI-CAR) with an activating CAR (aCAR) recognizing tumor antigens (ROR1 or CD19) and an inhibitory CAR (iCAR) targeting an NK-specific antigen (CS1) (Table 1; Figure 1B). When NK cells encounter their trogocytosed antigens, the inhibitory CAR sends out a “don’t-eat-me” signal, preventing fratricide, while the activating CAR ensures the effective recognition and elimination of tumor targets. Additionally, studies have shown that pretreatment of CAR-NK cells with immunological synapse inhibitors, such as Latrunculin A [18] and Cytochalasin D [19], prevents trogocytosis (Table 1; Figure 1B). While metabolic impairment following trogocytosis has been documented, strategies aimed at metabolic rescue have not yet been specifically investigated in detail in CAR-NK cells. However, studies in NK cells more broadly have examined metabolic dysregulation and potential modulatory pathways, which are discussed in more detail later in this review.

## 5. Addressing Tumor Stroma and Extracellular Matrix (ECM) Resistance

The TME consists of tumor cells, immunosuppressive immune cells, CAFs, complex stromal components, including ECM, basement membranes, and endothelial networks [85,86]. While the initial migration of immune cells towards the tumor targets is mediated via cytokine and chemokine gradients, their infiltration is substantially barricaded by the rigidity of the ECM [102]. CAFs, constituting 80% of the total tumor mass in certain malignancies [103], are the primary contributors of ECM deposition and TME remodeling. These cells secrete various mediators, including IL-6, PGE2, IDO, TGF-β, and matrix metalloproteinases (MMPs), all of which significantly impair NK cell cytotoxicity [20]. Administration of Nintedanib, an anti-fibrotic agent, significantly enhances CAR-NK tumor infiltration and cytotoxicity in pancreatic cancer models by suppressing CAF activation, increasing cancer cell killing from ~32% to ~42% (*p* < 0.0001) [21]. Engineering of CAR-NK cells targeting fibroblast activation protein (FAP), a serine protease overexpressed by CAFs (Figure 1C), has been shown to inhibit tumor progression in non-small cell lung cancer models [104] and cervical cancer models, where anti-FAP CAR-NK-92 cells demonstrated an 1.5-fold increase in CD107a expression and reduced CAF spheroid fluorescence by 50–83% within 34–72 h compared to controls (*p* < 0.05) [105]. Additionally, designing CAR-NK cells to secrete ECM-degrading enzymes such as heparanase [22], hyaluronidase [23,24], MMP [25], or collagenase [26] may overcome the stromal barriers and enhance CAR-NK penetration in the TME (Figure 1C). Several clinical trials are testing regional delivery of CAR-NK cells via the hepatic artery or intraperitoneally, which aligns with strategies to bypass stromal barriers (Table 2).

## 6. Enhancing CAR-NK Cell Infiltration and Homing to Tumors

Infiltration of NK cells is mediated via chemokine gradients generated by tumor, stromal, and immune cells within the TME; however, their infiltration remains suboptimal due to the chemokine system dysregulation [27]. Therefore, designing CAR-NK cells expressing tumor-matched chemokine receptors may enhance CAR-NK infiltration (Figure 1C). Studies reported that anti-mesothelin CAR-NK cells with CXCR2 showed enhanced pancreatic tumor infiltration [28], while NKG2D CAR-NK cells co-expressing CXCR1 showed improved migration and infiltration in xenograft models, resulting in a 20% increase in median survival compared to NKG2D CAR-NK cells lacking CXCR1 [29]. Similarly, EGFRvIII-targeting CAR-NK cells with CXCR4 demonstrated superior GBM infiltration and cytotoxicity in vivo, increasing intratumoral accumulation from 10–15 cells/field to around 35 cells/field [30]. Other chemokine receptors, such as CCR5 [31], CCR7 [32,106], and CCR4 [19], have been successfully engineered to enhance NK cell migration (Table 1).

Beyond genetic modification, chemokine receptor profiles can be drastically influenced during the ex vivo expansion of NK cells with specific cytokines. IL-2 and/or IL-15-mediated NK expansion upregulates CXCR3 expression [33,107]. While IL-2 alone upregulates CCR2 expression [34], combining it with glucocorticoids and IL-15 upregulates CXCR3 levels [108]. Finally, IL-18 in culture upregulates CCR7 expression [35]. Again, trogocytosis can also alter chemokine receptor expression; co-culturing of NK cells with CCR7^+^ K562 cells can lead to the transfer of CCR7 on NK cells, thereby facilitating their migration towards CCL19 and CCL21 ligands [109] (Table 1).

Trafficking of CAR-NK cells can be further enhanced through combination therapies and sophisticated delivery methodologies designed to optimize the TME for lymphocyte recruitment [110]. Approaches to modify the TME include using immunomodulatory agents, such as oncolytic viruses [36] or radiotherapy [25], which can boost tumor inflammation, stimulate immune responses, and improve CAR-NK cell efficacy. Finally, local injection of CAR-NK cells to the tumor sites is a strategic delivery route that has consistently demonstrated to be both safe and effective in preclinical and clinical investigations [37,38] (Table 1 and Table 2). Current clinical studies adopting local delivery/injection routes to enhance trafficking and exposure are listed in Table 2.

## 7. Modulating Hypoxia and Immunosuppressive Factors in the TME

Tumor hypoxia critically impairs NK cell function, where the upregulation of hypoxia-inducible factors (HIFs), comprising oxygen-sensitive alpha subunits (HIF-1α, HIF-2α, HIF-3α) and constitutive beta subunits (HIF-1β), triggers detrimental cellular changes [39]. HIF-1α downregulates activating ligands, like MICA and MICB, on tumor cells through MMP-mediated shedding [111] and suppresses the expression levels of critical NK activation markers, including NKG2D, NKp30, NKp44, and NKp46 [40], resulting in reduced NK cell cytotoxicity. It further suppresses the production of the degranulation marker CD107a and the secretion of cytotoxic molecules, including granzyme B and perforin, by inhibiting the phosphorylation of extracellular signal-regulated kinases and signal transducer and activator of transcription 3 [41]. Therefore, blocking HIF-1α signaling in NK cells has been shown to improve their anti-tumor efficacy [44]. Furthermore, therapeutic agents targeting HIFs, such as those that inhibit their synthesis, dimerization formation, or trigger degradation, are currently being evaluated [112,113] with the potential to be integrated with CAR-NK therapy (Figure 1E). As shown in CAR-T cell studies, engineering CAR-NK cells with a hypoxia-response element [114,115] or oxygen-sensitive subdomains derived from HIF-1α [116,117] only ensures specificity in hypoxic conditions and reduces off-target effects in normoxic environments (Table 1; Figure 1E).

Hypoxic conditions force NK cells to switch to glycolysis from oxidative phosphorylation, which limits their energy availability and impairs their cytotoxicity. The shift towards glycolysis leads to the accumulation of lactate, which suppresses IFN-γ production, downregulates the expression of NKp46, CD107a, and granzyme B, and triggers mitochondrial damage and NK cell apoptosis [42]. Currently, glycolytic inhibitors and lactate dehydrogenase blockers that may counteract these metabolic challenges are being investigated (Figure 1E) [43]. Another way to improve the metabolic dysregulation is simultaneously integrating metabolic regulators, including adenosine monophosphate-activated protein kinase (AMPK) and peroxisome proliferator-activated receptor gamma coactivator 1-alpha (PGC-1α), which enhances mitochondrial function and oxidative phosphorylation capacity [118]. While strategies aimed at metabolic rescue have been primarily investigated in NK cells, there remains a research gap in exploring these approaches within CAR-NK cells. Hypoxia also impairs NK cell functionality by modulating the immunosuppressive cells, such as regulatory T cells, M2 TAMs, and MDSCs, within the TME. CAR-NK cells have been engineered to directly target these immunosuppressive populations, particularly MDSCs [119] and TAMs [120], to enhance their anti-tumor activity (Table 1).

Hypoxia-driven expression of CD39 and CD73 by tumor and regulatory T cells elevates adenosine levels, which further reduces NK cell activity via the adenosine A2A receptor [45]. Blocking of CD73 with antagonist antibodies has been shown to improve CAR-NK efficacy (Figure 1E) [46]. Chambers and colleagues [47] also showed that engineering CAR-NK cells targeting CD73 not only exhibited improved infiltration and cytotoxicity against CD73^+^ tumor cells but also significantly reduced adenosine production (~0 µM), lowering it below the baseline levels observed in cancer cells cultured alone. Furthermore, deletion of the A2A receptor via genetic editing has shown enhanced CAR-NK mediated killing in preclinical models [48,121,122] (Table 1; Figure 1E).

The secretion of TGF-β from the immunosuppressive cells within the TME downregulates the expression of NK activation receptors NKG2D, DNAM-1, and NKp30 and reduces IFN-γ production [49]. Multiple strategies have been explored to block the immunosuppressive effects of TGF-β, including the silencing of TGFβ-induced miR-27a-5p in vivo [50], the deletion of the TGF-β receptor (TGFβR)-2 [51], and the administration of the TGF-β receptor kinase inhibitor [52], all of which led to enhanced NK cell functionality (Figure 1E). Furthermore, by engineering CAR-NK cells with high affinity, dominant-negative TGF-β receptors have been shown to resist the suppressive effects of TGF-β while maintaining their therapeutic potency [53,123]. TGF-β also reduces NK cell activity via the production of reactive oxygen species (ROS) [54]. Addressing this issue, Liu and colleagues [55] designed a CAR-NK cell targeting HER1 to co-express catalase that successfully neutralized the accumulated ROS hydrogen peroxide in the TME and enhanced CAR-NK cell functionality (Table 1; Figure 1E).

Hypoxia-induced loss of NK cytotoxicity can also be rescued by treating CAR-NK cells with cytokines, like IL-2, IL-15, IL-18, and IL-21, which improve survival, proliferation, and cytotoxic activity [44,56,124,125,126,127]. Additionally, BiKEs and TriKEs have been designed to further boost CAR-NK cell activity by simultaneously targeting tumor antigens and engaging CD16 on NK cells, with TriKEs additionally delivering IL-15 to support NK cell persistence and proliferation [56,128]. These approaches largely benefit CAR-NK anti-tumor efficacy, as the activation of BiKEs and TriKEs depends on CD16, and hypoxia has been shown not to impact CD16-mediated antibody-dependent cellular cytotoxicity [125]. Furthermore, research is being conducted on novel NK cell engagers that can activate receptors, like NKp46, NKp30, and NKG2D, which are significantly suppressed under hypoxic conditions [129,130] (Table 1).

## 8. Improving NK Cell Persistence and Survival

The inherent short lifespan and high turnover rates in vivo constrain NK cell persistence in solid tumors compared to other lymphocytes [131]. Their sustained functionality is dependent on the maintenance of the homeostatic balance between activating and inhibitory signaling, as overstimulation often leads to NK cell exhaustion [132]. This can be reduced by blocking the inhibitory receptors such as KIRs, PD-1, TIGIT, NKG2A, and TIM-3 (Figure 1D) [84,133]. Extensive research is being carried out investigating various checkpoint blockade therapies targeting NK cell inhibitory receptors (Section 9).

Another strategy to boost CAR-NK cell proliferation, persistence, and survival is by supplementing various cytokines such as IL-2, IL-12, IL-15, and IL-18. Armoring CAR-NK cells with IL-15 in their construct enhances their in vivo persistence and improves their metabolic fitness, leading to enhanced anti-tumor activity (Figure 1D) [57,126]. Another strategy to enhance their persistence is by targeting the negative regulators of cytokine signaling, such as the cytokine-inducible SH2-containing protein (CIS). CIS is a negative regulator of both IL-2 and IL-15 signaling, and knocking out of the cytokine-inducible SH2-containing protein gene (*CISH*) using clustered regularly interspaced short palindromic repeats (CRISPR)/CRISPR-associated nuclease 9 (Cas9) in induced pluripotent stem cell-derived CAR-NK cells have been shown to significantly improve metabolic fitness through enhanced mitochondrial activity and mTOR signaling, resulting in overall improvement of NK performance and persistence (Figure 1D) [58]. Additionally, co-expressing anti-apoptotic genes, such as BCL-2 or BCL-XL, may also improve CAR-NK cell survival (Figure 1D) [59]. Another approach to ensure enhanced metabolic adaptation of CAR-NK cells within the TME is to increase the essential amino acid uptake. Increased expression of specific amino acid transport molecules, particularly SLC1A5 and the SLC3A2/SLC7A5 transporter complex, facilitates growth, proliferation, and persistence of CAR-NK cells (Table 1) [60].

## 9. Incorporating Immune Checkpoint Inhibitors

### 9.1. PD-1

Immune checkpoint inhibitors play a crucial role in regulating persistent immune responses. In the TME, the tumor cells exploit immune checkpoint pathways to suppress NK cell function, predominantly through the PD-1/PD-L1 axis (Figure 1F). PD-1 is abundantly expressed on activated NK cells, and upon ligation with the PD-L1 receptor overexpressed in tumor cells, leads to immunosuppression, thereby impairing NK cell functionality [134]. To counteract this, CAR-NK cells have been engineered with chimeric costimulatory converting receptors that convert the PD-1 inhibitory signals into activation. Combining the extracellular domain of PD-1 with NKG2D signaling components, along with the 4-1BB co-stimulatory domain, has demonstrated robust in vitro cytotoxicity in various cancer cell models [61] and induced significant gasdermin E-dependent pyroptosis in lung cancer models [62]. A dual CAR incorporating PD-1 and NKG2D recognition domains with DAP10 signaling showed an approximate 27% and 50% increase in TNF-α and TNF-related apoptosis-inducing ligand (TRAIL) production, respectively, enhanced cytotoxicity, and triggered apoptosis in gastric cancer cells [63]. Another innovative engineering of CAR-NK-92, combining IL-15Rα-sushi/IL-15 complexes with PD-1 signal inverters, demonstrated superior persistence and cytotoxicity against pancreatic cancer cells [64]. Additionally, using CRISPR/Cas9 to KO inhibitory genes, such as a disintegrin and metalloproteinase-17 (ADAM-17) and PD-1, enhances NK cell cytotoxicity and cytokine production [65] (Table 1).

### 9.2. PD-L1

Similarly, CAR-NK cells targeting PD-L1 have demonstrated enhanced cytotoxicity across multiple solid tumor models. High-affinity NK (haNK) cells engineered NK-92 cells expressing endoplasmic reticulum-retained IL-2, with PD-L1 CAR effectively recognized and eliminated various tumor targets, including triple-negative breast cancer and lung, urogenital, gastric, and head and neck squamous cell carcinoma [66,135]. Furthermore, these engineered CAR-NK cells significantly reduced immunosuppressive macrophages and MDSCs expressing high PD-L1 levels [66,135]. Integrating these PD-L1 haNK cells with anti-PD1 and N-803, an IL-15 super agonist, demonstrated superior tumor growth control in preclinical models [66]. Fourth-generation PD-L1 CAR-NK cells co-expressing IL-15 further enhanced the persistence and functionality of CAR-NK cells against various tumor cell lines [68] (Table 1).

In heterogeneous tumor models, PD-L1 CAR-NK cells successfully eliminated resistant populations that escaped T cell-mediated killing due to antigen presentation defects and PD-L1 upregulation [67]. A novel atezolizumab-based PD-L1 CAR-NK cell showed potent activity against tumors overexpressing PD-L1 and exhibited a self-amplifying effect by inducing PD-L1 expression on PD-L1 low tumor cells, thereby enhancing cytotoxicity [69]. CAR-NK cells derived from hematopoietic progenitor cells and human pluripotent stem cells targeting PD-L1 showed enhanced anti-tumor activity both in vitro and in vivo. In addition to lysing tumors expressing high levels of PD-L1, they also revived exhausted T cells, thereby promoting a stronger immune response [136,137]. Dual CAR-NK cells targeting PD-L1 and folate receptor alpha showed enhanced cytotoxicity and promoted the development of a memory-like phenotype in NK cells [138]. Similarly, another dual CAR-NK construct targeting PD-L1 and HER2 demonstrated superior therapeutic efficacy against multiple solid tumors, including breast, ovarian, pancreatic, and gastric cancer cell lines, while minimizing immune escape through antigen loss [7] (Table 1).

Lastly, combining PD-L1 CAR-NK cells with immune checkpoint inhibitors may further enhance anti-tumor activity, as reported in CAR-T studies involving engineered CARs to secrete anti-PD-1 scFvs [70], co-transduction with PD-1 short hairpin ribonucleic acid (RNA) [71], or adenine base editing to downregulate PD-1 expression [72]. All these approaches reduced exhaustion and improved the cytotoxic function of CAR-T cells, and similar strategies can be explored in CAR-NK therapies (Table 1).

### 9.3. Human Leukocyte Antigen-G (HLA-G)

HLA-G is a non-classical major histocompatibility complex (MHC) class I molecule that binds with immunoglobulin-like transcript 2 (ILT2) and killer cell immunoglobulin-like receptor (KIR) DL4 on NK cells. This triggers an immunosuppressive microenvironment by encouraging the expansion of immunosuppressive cells, like regulatory T cells and MDSCs, and by diminishing NK cell functionality, resulting in immune evasion of tumor cells [73]. HLA-G targeting CAR-NK cells with a DAP12 intracellular signaling domain demonstrated significant cytotoxicity both in vitro and in vivo against several solid tumor models, including breast, brain, pancreatic, and ovarian cancer cells (Figure 1F) [74]. Co-culture of the anti-HLA-G CAR-NK cells with tumor cells led to the activation of the Syk/Zap70 signal transduction cascade of NK cells, thereby reversing the HLA-G-mediated immunosuppressive signals [74] (Table 1).

### 9.4. B7-H3 (CD276)

The overexpression of the B7 homologous 3 protein (B7-H3) immune checkpoint molecule in several cancers has been shown to suppress NK-mediated cell lysis [139]. Anti-B7-H3 CAR-NK-92 cells demonstrated significantly higher cytotoxic potential and elevated cytotoxic markers, including perforin, granzyme B, and CD107a, against tumor targets both in vitro and in vivo (Figure 1F) [75]. Similarly, Grote and colleagues [76] developed a second-generation B7-H3 CAR-NK-92 cell line, which showed high specificity by lysing neuroblastoma cells expressing high B7-H3 levels while sparing B7-H3-negative cell populations. The same study investigated the resilience of the engineered CAR-NK cells against the TME challenges, including low pH, hypoxia, and diverse immunosuppressive factors. The B7-H3 CAR-NK cells exhibited remarkable therapeutic potential by overcoming the TME-mediated immunosuppression and successfully lysing melanoma cells [76] (Table 1).

### 9.5. CD47/Signal Regulatory Protein Alpha (SIRPα)

CD47 is an inhibitory ligand overexpressed in multiple cancer cells. It binds with SIRPα on innate immune cells, which triggers “don’t-eat-me” signaling, ultimately leading to tumor immune evasion. This interaction suppresses macrophage phagocytosis [77] and NK cell cytotoxicity against tumor cells [78]. Additionally, elevated CD47 messenger RNA levels correlate with poor patient survival outcomes across multiple solid malignancies [77,79]. Therefore, various approaches have been investigated to disrupt this CD47/SIRPα axis, including blocking antibodies, recombinant SIRPα proteins, and strategic CAR designs. Anti-CD47 antibodies combined with CAR-NK cells demonstrated robust anti-tumor activity in gastrointestinal cancer models [80]. While limited studies are available on anti-CD47 CAR-NK cells, CAR-T cells targeting CD47 show great efficacy against ovarian [81], pancreatic [140], lung [141], and osteosarcoma models (Figure 1F). [82]. CAR immune cells have also been engineered to secrete soluble CD47/SIRPα-blocking agents, including chimeric monoclonal CD47 antibodies [142], SIRPα-Fc fusion proteins [83], and high-affinity CD47 blockers, like CV1 [80,143] and SIRPγ-derived protein (SGRP) [144]. Disrupting the CD47-SIRPα axis enables macrophage phagocytosis, induces phenotype switch from M2 to M1 TAMs [77,83,145], reduces CAR cell therapy resistance, and improves NK cell anti-tumor activity [78] (Table 1). Clinically, CD47 targeting is constrained by on-target, off-tumor toxicity since CD47 is expressed on normal cells, posing safety risks. For example Magrolimab, a monoclonal antibody that blocks CD47, causes anemia in patients with myelodysplastic syndrome [146].

## 10. Clinical Translation and Ongoing Trials

To highlight the strategies reviewed above, we compiled a table of current CAR-NK clinical trials in solid tumors, summarizing CAR constructs, targets, and phases (Table 2). These trials show first-in-human studies targeting TROP2, PD-1, PD-L1, NKG2D, and others as indicated, with several testing intraperitoneal delivery, hepatic artery transfusion, or combinations with checkpoint blockades and cytokine agonists. A phase II trial of irradiated PD-L1 CAR-NK plus pembrolizumab and N-803 in gastroesophageal and head and neck cancers (NCT04847466, Table 2) reflects emphasis on using CAR-NK cells with immune modulation. Additionally, TROP2 CAR-NK studies, including IL-15 transduced NK cells, aim to enhance persistence, and NKG2D CAR-NK cells may mitigate antigen heterogeneity by recognizing multiple stress ligands (Table 2). In solid tumors, both CAR-T and CAR-NK therapies remain largely confined to early phase I/II studies with modest response rates compared with hematological malignancies. Direct comparisons between CAR-T and CAR-NK in the same indications are not yet available, and cross-trial comparisons are complicated by differences in targets, constructs, and trial design. At present, the rationale for prioritizing CAR-NK engineering in solid tumors rests mainly on their favorable safety profile, off-the-shelf potential, and encouraging preclinical data in TME-relevant models rather than on proven clinical superiority over CAR-T cells [147].

## 11. Manufacturing Challenges and Limitations of CAR NK Therapy

Despite showing fewer severe toxicities than CAR-T cells due to their shorter lifespan, limited in vivo expansion, and lower cytokine secretion, CAR NK cells still possess some limitations. Off-target binding and on-target, off-tumor toxicity remain a major concern, as few tumor-associated antigens are exclusively expressed on malignant cells. Therefore, it is crucial to develop CAR constructs that can strictly discriminate between malignant and healthy cells. Emerging computation and AI-based antigen discovery tools may help design scFvs with greater tumor specificity while reducing off-target cytotoxicity. Additionally, to improve their persistence, CAR NK cells are often armored with cytokines, such as IL-15, which may lead to systemic toxicity, immune dysregulation, or uncontrolled proliferation [148]. The most common side effects reported after CAR NK transfusion include fever and fatigue, arising from the increase in IL-6 and C-reactive protein [1]. To address these concerns, inducible or logic-gated CAR NK cells (“on-switch” AND/OR-gated CARs) have been designed to control unintended activation or include safety switches, such as inducible caspase-9, that allow selective depletion of CAR-NK cells in cases of severe toxicity [149].

Although CAR NK therapy has shown promising results in preclinical and early clinical studies, clinical-scale manufacturing of CAR NK cells remains a significant barrier to widespread adoption, largely due to the lack of standardization. Various allogeneic sources have been used to generate CAR NK cells, including peripheral blood, umbilical cord blood, NK cell lines (such as NK-92), and iPSCs, each with distinct advantages and limitations previously reviewed in detail [150]. The diverse sources of NK cells, while advantageous, introduce production challenges as different sources exhibit highly varying transduction and expansion efficacy, persistence, and cytotoxic activity, leading to varying performances of the product. Furthermore, unlike other cells, the transduction and transfection efficiency is much lower in NK cells [151], therefore making it much more difficult to generate high levels of genetically modified NK cells. In addition, the batch variability in titer and purity often seen in clinical-grade lentiviral and retroviral vectors ultimately impacts the gene transduction efficiency [152]. Moreover, gene transduction via viral vectors may lead to insertional mutagenesis, which poses the risk of genome instability and secondary malignancies [153]. iPSC-derived NK cells address many of these limitations by providing a renewable, genetically defined, and clonally stable source. iPSCs can be precisely genetically engineered at the pluripotent stage using CRISPR-Cas9 and then cloned to generate uniform, fully functional NK cells with stable CAR expression, therefore eliminating the need to virally transduce mature NK cells. Moreover, these cloned, genetically edited iPSCs allow the generation of CAR-engineered master cell banks that can be expanded, differentiated into defined effector NK cells, cryopreserved, and distributed as standardized “off-the-shelf” products, greatly reducing production time, cost, and variability while ensuring improved persistence and cytotoxicity [154]. The lack of a standardized CAR design, along with significant variation in genetic engineering methods, vector systems, expression strategies, and NK cell sources across different research institutions, further adds to the challenges for clinical application. Therefore, further research determining which genetic engineering approaches and combinations work best for producing effective CAR-NK therapies is required. While advanced genetic engineering strategies, such as quadruple gene-engineered CAR NK cells, have shown durable anti-tumor activity in preclinical studies [155], each genetic modification further adds to the manufacturing complexity. Finally, generating consistent product quality under Good Manufacturing Practice (GMP) conditions requires strict control of GMP-grade cytokines, serum-free media, and seed cell thawing protocols. Feeder-free expansion systems in bioreactors often fail to achieve yields comparable to feeder-based protocols while maintaining regulatory compliance [156]. Furthermore, cryopreservation impairs CAR NK cell activity [157]; therefore, research addressing this and post-thaw viability is crucial. For off-the-shelf use, it is essential to develop a standardized, scalable, and reproducible manufacturing pipeline ensuring the safety, efficacy, and accessibility of next-generation CAR-NK therapies.

## 12. Conclusions

Effective solid tumor control with CAR-NK therapy will require addressing TME barriers (summarized in Figure 1). This consists of broadening target recognition, preventing antigen loss, limiting fratricide, overcoming stromal barriers, hypoxia, and metabolic stress, enhancing homing, sustaining cell persistence, and blocking inhibitory checkpoints. Early clinical data support the feasibility of these strategies, including multi-antigen targeting, engineered cytokine support, and regional delivery of CAR-NK cells. The future of CAR NK therapy lies in addressing these limitations using innovative approaches and combination strategies. Artificial intelligence has been integrated into CAR therapy, where deep learning approaches enable multi-antigen targeting and antigen escape prevention, natural language processing facilitates clinical data extraction from reports and the literature, computer vision analyzes CAR cell morphology and phenotypes, reinforcement learning optimizes dosing schedules, and predictive algorithms assess therapeutic efficacy and toxicity profiles [158]. Although emerging artificial intelligence and machine learning tools may aid CAR-NK design in the future, their current use in solid tumors is still exploratory.

Prioritizing CAR-NK combinations should be driven by the main barrier in each tumor. In desmoplastic, stroma-rich cancers, pairing CAF/ECM targeting or anti-stroma approaches with multi-antigen targeting may best improve both access and specificity. In hypoxic TMEs, combinations that couple metabolic or mitochondrial support (for example, cytokine armoring or metabolic re-programming) with persistence tools and checkpoint modulation may provide impact, whereas in checkpoint or soluble factor-driven settings, multi-antigen targeting, checkpoint blockade, and cytokine support may provide better efficacy. Additionally, combination strategies such as administering immune checkpoint inhibitors, kinase inhibitors, protease inhibitors, chemotherapy, radiotherapy, STING agonists, oncolytic viruses, and photothermal therapy that can regulate or reshape the TME present opportunities to improve CAR NK homing, persistence, and cytotoxicity [159].

## Figures and Tables

**Table 1:** Summary of the major TME barriers and resistance mechanisms impairing CAR-NK cell function, the corresponding engineering strategies to reduce them, and their reported outcomes, which are all derived from preclinical studies *.

TME Barrier/Resistance Mechanism	CAR-NK Impairment in the TME	CAR-NK Engineering Strategy	Outcome	References
Antigen heterogeneity and immune escape	Loss of, or low density of TAAs, reduces CAR engagement; heterogeneous tumors evade recognition	Higher affinity scFvs (using directed evolution, computational modeling, and phage display); multi-targeting CARs; multi-specific engagers	Enhanced recognition and killing of low antigen tumors; reduced escape; improved efficacy (IL-15 in TriKEs)	[2,3,4,5,6,7,8,9,10,11,12,13]
Signaling insufficiency in CAR design	Suboptimal activation/costimulation	NK cell-specific signaling domains (2B4, DNAM-1, NKG2D); adaptor-coupled signaling (DAP10/DAP12)	Improved activation and cytotoxicity	[14,15]
Trogocytosis-driven antigen loss and fratricide	CAR-NK exhaustion (upregulated LAG-3/TIM-3); metabolic dysregulation (downregulated glycolysis)	Dual AI-CAR to deliver self-recognition “don’t-eat-me” signals; pretreat with synapse inhibitors (Latrunculin A, Cytochalasin D)	Prevents fratricide; preserves CAR density; decreases trogocytosis	[16,17,18,19]
Stroma/ECM rigidity and CAF-mediated suppression	Physical exclusion; soluble suppressors (IL-6, PGE2, IDO, TGF-β, MMPs) impair cytotoxicity	Anti-fibrotic agents (Nintedanib); FAP-CAR NK to target CAFs; CAR-NK secreting ECM-degrading enzymes	Improved tumor infiltration; reduced tumor growth; increased CAR-NK killing in solid tumor models	[20,21,22,23,24,25,26]
Chemokine dysregulation limits NK trafficking	Poor homing/infiltration to tumor beds	Engineer tumor matched chemokine receptors (CXCR1/2/4, CCR4/5/7); tune expansion cytokines (IL-2/15/18) to shape receptor profiles; oncolytic viruses/radiotherapy to condition TME; local delivery	Enhanced migration and infiltration; improved cytotoxicity and tumor control in xenografts; safe and effective local injection	[19,25,27,28,29,30,31,32,33,34,35,36,37,38]
Hypoxia (HIF-driven) and metabolic stress (lactate, nutrient depletion)	↓NKG2D/NKp30/NKp44/NKp46; ↓CD107a, perforin, granzyme B, IFN-γ; mitochondrial damage; apoptosis	Block HIF-1α; glycolysis/lactate dehydrogenase blockade; metabolic rewiring (AMPK/PGC-1α); hypoxia-responsive CAR control elements	Restored activation and degranulation; improved specificity to hypoxic TMEs; enhanced survival/effector function	[39,40,41,42,43,44]
Hypoxia-induced modulation of the adenosine axis (CD39/CD73 via A2A receptor)	A2A receptor signaling suppresses NK cell activity	Anti-CD73 antibodies or CD73 CAR-NK; A2A receptor deletion	Lower adenosine in the TME; increased CAR-NK infiltration and cytotoxicity	[45,46,47,48]
TGF-β-mediated suppression and ROS	↓NKG2D/DNAM-1/NKp30; ↓IFN-γ; ROS-reduced NK cell activity	TGF-β pathway inhibition; dominant-negative, CAR-NK with high-affinity TGFβR; co-expression of catalase to detoxify H_2_O_2_	Resistance to TGF-β; improved cytotoxicity and cytokine production; better function within the oxidative TME	[49,50,51,52,53,54,55]
Limited persistence/survival of NK cells	Short lifespan; exhaustion from chronic stimulation; poor metabolic fitness	Treatment with cytokines; armoring (IL-15 ± IL-12/18/21); *CISH* knockout (KO); anti-apoptotic genes (BCL-2/BCL-XL); increase essential amino acid transport (SLC1A5/SLC3A2/SLC7A5 complex)	Improved proliferation, metabolic fitness (↑mitochondria, mTOR), persistence, and anti-tumor activity	[56,57,58] [59,60]
PD-1/PD-L1 axis	Inhibitory signaling via PD-1/PD-L1 reduces NK effector function	PD-1 signal converter CAR-NK; PD-1 or ADAM-17 KO; PD-L1-CAR NK (±IL-15), combinations with immune checkpoint inhibitors/N-803; dual CARs (PD-L1 + HER2)	Increased cytotoxicity, pyroptosis/apoptosis of tumor cells; reduced suppressive myeloid cells; better control of heterogeneous tumors	[7,61,62,63,64,65,66,67,68,69,70,71,72]
Other checkpoints (HLA-G, B7-H3)	ILT2/KIR2DL4 (HLA-G) and B7-H3 signaling dampen NK function	HLA-G CAR NK with DAP12; B7-H3 CAR-NK	Potent in vitro/in vivo cytotoxicity across multiple solid tumor models; resilience to low pH/hypoxia/suppressive factors	[73,74,75,76]
CD47/SIRPα axis	Macrophage phagocytosis blocked; NK function reduced; poor patient survival outcomes correlate with high CD47	Block CD47/SIRPα with antibodies or soluble blockers (SIRPα-Fc, CV1); CD47 CAR-NK; engineer CAR cells to secrete blocking agents	Restored innate immunity (↑phagocytosis, M2→M1); improved CAR-NK efficacy and resistance	[77,78,79,80,81,82,83]

* Abbreviations are defined at the end of the manuscript; ↓ downregulation/suppression; ↑ upregulation.

**Table 2:** Current CAR-NK trials in solid tumors *.

Solid Tumor	CAR Structure	Target	Phase	NCT Number	Study Title
Advanced head and neck cancer	TROP2 CAR/IL-15 TGFβR-2 KO NK	TROP2	I	NCT07101432	Phase I study of preconditioning radiation therapy with IL-15 transduced TGFBR2 KO CAR.TROP2-engineered cord blood-derived NK cells in patients with advanced head and neck cancer (RADIANCE-NK)
Clear cell carcinoma	TGFβR-2 KO CD70 CAR NK	CD70	I	NCT07072234	Phase I study of allogeneic transforming growth factor-beta receptor type 2 KO CD70 CAR NK cells in treatment refractory clear cell renal cell carcinoma
Advanced gastric or colorectal cancer	PD-1-M1-NK	PD-1	I	NCT07031011	An early clinical study to investigate the safety, pharmacokinetics, and efficacy of PD-1-M1-NK cells (YC-T-001) in patients with advanced gastric or colorectal cancer failed or intolerant to at least second-line therapy
Advanced solid tumors with liver metastases	NKG2D CAR-NK	NKG2D ligands	I	NCT07021534	Hepatic artery transfusion of NKG2D CAR-NK cells followed by intravenous infusion of NKG2D CAR-T Cells to treat patients with advanced solid tumors with liver metastases who have failed standard treatments: a phase I exploratory clinical trial
Pancreatic cancer	CL-NK-001	N/A	I	NCT06816823	A clinical study of CAR-NK Cells (CL-NK-001) in patients with advanced pancreatic cancer
Advanced solid tumors	Universal NK cell (NK042)	N/A	I	NCT06773091	An open-label, single-arm, multicenter phase I clinical study to evaluate the safety and preliminary efficacy of NK042 cell injection (Universal NKR + NK) in advanced solid tumors
Hepatocellular carcinoma	SN301A CAR NK	GPC3	I	NCT06652243	An early clinical study to evaluate the safety and efficacy of SN301A cell injection in the treatment of subjects with Glypican-3 (GPC3)-positive advanced hepatocellular carcinoma
Pancreatic cancer non-resectable	NKG2D CAR NK	NKG2D ligands	I	NCT06503497	A single-center, single-arm, open-label, dose-escalation clinical study to evaluate the safety and anti-tumor efficacy of second-line systemic chemotherapy sequential NKG2D CAR-NK cell therapy for pancreatic cancer
Pancreatic cancer non-resectable	NKG2D CAR NK	NKG2D ligands	I	NCT06478459	A single-center, single-arm, open-label, dose-escalation clinical study to evaluate the safety and anti-tumor efficacy of intratumoral NKG2D CAR-NK cell injection guided by EUS in the treatment of locally advanced pancreatic cancer
Gastric cancer, pancreatic adenocarcinoma	CB CAR-NK182 cell	Claudin18.2	I	NCT06464965	A phase I clinical study of cord blood-derived CAR-NK cells targeting Claudin18.2 in the treatment of advanced gastric cancer and advanced pancreatic cancer
Non-small cell lung cancer	Anti-TROP2 universal CAR-NK	TROP2	I/II	NCT06454890	An investigator-initiated trial evaluating the efficacy and safety of anti-TROP2 universal CAR-NK(U-CAR-NK) cells therapy combined with chemotherapy for relapsed/refractory non-small cell lung cancer (NSCLC)
Colorectal cancer with minimal residual disease	TROP2 CAR-NK	TROP2	I	NCT06358430	Phase I dose escalation and expansion study of TROP2 CAR engineered IL-15-transduced cord blood-derived NK cells in combination with cetuximab in patient with colorectal cancer (CRC) with minimal residual disease (MRD)
Solid tumors	TROP2 CAR-NK	TROP2	I	NCT06066424	Phase I dose escalation and expansion study of TROP2 CAR engineered IL15-transduced cord blood-derived NK cells in patients with advanced solid tumors (TROPIKANA)
Pancreatic, ovarian, adenocarcinoma	TROP2 CAR/IL15-transduced CB-NK	TROP2	I/II	NCT05922930	Phase I/II study of TROP2 CAR engineered IL15-transduced cord blood-derived NK cells delivered intraperitoneally for the management of platinum resistant ovarian cancer, mesonephric-like adenocarcinoma, and pancreatic cancer
Ovarian epithelial carcinoma	SZ011 CAR-NK	SZ011	I	NCT05856643	Single-arm, open-label clinical study of SZ011 in the treatment of ovarian epithelial
Ovarian cancer	NKG2D CAR-NK	NKG2D ligands	N/A	NCT05776355	NKG2D CAR-NK cell therapy for patients with platinum-resistant recurrent ovarian cancer
Advanced renal cell carcinoma, mesothelioma, and osteosarcoma	CAR.70/IL15-transduced CB-derived NK	CD70	I/II	NCT05703854	Phase I/II study of CAR.70-engineered IL15-transduced cord blood-derived NK cells in conjunction with lymphodepleting chemotherapy for the management of advanced renal cell carcinoma, mesothelioma and osteosarcoma
Triple-negative breast cancer	SZ011 CAR NK	SZ011	I	NCT05686720	Single-arm, open-label clinical study of SZ011 in the treatment of advanced triple negative breast cancer
Relapsed/refractory extensive-stage small cell lung cancer	DLL3 CAR-NK	DLL3	I	NCT05507593	A multicenter phase I trial on the safety and preliminary efficacy of DLL3 CAR-NK cells in the treatment of relapsed/refractory extensive stage small cell lung cancer
Stage IV ovarian cancer, testis cancer refractory, endometrial cancer recurrent	Claudin6, GPC3, mesothelin, or AXL targeting CAR-NK	Claudin6, GPC3, mesothelin, or AXL	I	NCT05410717	Phase I trial to evaluate safety and preliminary efficacy of CLDN6/GPC3/Mesothelin/AXL-CAR-NK in patients with CLDN6/GPC3/Mesothelin/AXL-positive advanced solid tumors
Refractory metastatic colorectal cancer	NKG2D CAR-NK	NKG2D ligands	I	NCT05213195	NKG2D CAR-NK cell therapy in patients with refractory metastatic colorectal cancer
Advanced solid tumors	Anti-5T4 CAR-NK	5T4	I	NCT05194709	Clinical trial of anti-5T4 oncofetal trophoblast glycoprotein (5T4) conjugated antibody redirecting natural killer (CAR-NK) cells in advanced solid tumors
Gastroesophageal junction cancers, advanced head and neck squamous cell carcinoma	PD-L1 CAR-NK	PD-L1	II	NCT04847466	A Phase II study of immunotherapy combination: Irradiated PD-L1 CAR-NK cells plus pembrolizumab plus N-803 for subjects with recurrent/metastatic gastric or head and neck cancer
Pancreatic cancer	ROBO1 CAR-NK	ROBO1	I/II	NCT03941457	Clinical research of ROBO1 specific BiCAR-nk cells on patients with pancreatic cancer
Solid tumors	ROBO1 CAR-NK	ROBO1	I/II	NCT03940820	Clinical research of ROBO1 specific CAR-NK Cells on patients with solid tumors
Malignant tumors	BiCAR-NK/T cells (ROBO1 CAR-NK/T cells)	ROBO1	I/II	NCT03931720	Clinical research of ROBO1 specific CAR-NK Cells on patients with malignant tumor
Metastatic castration-resistant prostate cancer	Anti-PSMA CAR NK	PSMA	I	NCT03692663	Clinical study on the safety and efficacy of anti-PSMA CAR NK cells in metastatic castration-resistant prostate cancer (mCRPC)
Epithelial ovarian cancer	Anti-mesothelin CAR NK	Mesothelin	I	NCT03692637	Clinical study on the safety and efficacy of anti-mesothelin CAR NK cells with epithelial ovarian cancer
Solid tumors	NKG2D ligand targeted CAR-NK	NKG2D ligands	I	NCT03415100	Pilot study of NKG2D-ligand targeted CAR-NK Cells in patients with metastatic solid tumours
Advanced refractory or relapsed solid tumor	Anti-MUC1 CAR-pNK cells	MUC1	I/II	NCT02839954	Study evaluating the efficacy and safety of chimeric antigen receptor-modified pNK cells in MUC1 positive advanced refractory or relapsed solid tumor

* Abbreviations are defined at the end of the manuscript.

## Data Availability

No new data were created or analyzed in this study. Data sharing is not applicable to this article.

## Abbreviations

The following abbreviations are used in this manuscript:

2B4	CD244; SLAM family co-receptor on NK and T cells
5T4	oncofetal trophoblast glycoprotein
A2A	adenosine A2A receptor
ADAM	a disintegrin and metalloproteinase
AI-CAR	dual CAR (activating ROR1/CD19 CAR and CS1 inhibitory CAR)
AMPK	adenosine monophosphate-activated protein kinase
AXL	AXL receptor tyrosine kinase
B7-H3	B7 homolog 3
BCL-2	B cell lymphoma 2
BCL-XL	B cell lymphoma-extra large
BiKE	bispecific killer engager
CAFs	cancer-associated fibroblasts
CAR	chimeric antigen receptor
Cas9	CRISPR-associated nuclease 9
CCR	CC motif chemokine receptor
CD	cluster of differentiation
CIS	cytokine-inducible SH2-containing protein
*CISH*	cytokine-inducible SH2-containing protein gene
CRISPR	clustered regularly interspaced short palindromic repeat
CXCR	CXC motif chemokine receptor
DAP10, 12	adaptor proteins for activating NK receptors
DLL3	delta-like ligand 3
DNAM-1	DNAX accessory molecule 1
ECM	extracellular matrix
EGFR	epidermal growth factor receptor
FAP	fibroblast activation protein
GD2	disialoganglioside GD2
GBM	glioblastoma
GPC3	glypican-3
haNK	high-affinity NK cell
HER2	human epidermal growth factor receptor 2
HIFs	hypoxia-inducible factors
HLA-G	human leukocyte antigen-G
ICD	intracellular domain
IDO	indoleamine 2,3-dioxygenase
IFN	interferon
IL	interleukin
ILT2	immunoglobulin-like transcript 2
KIR	killer cell immunoglobulin-like receptor
KO	knockout
LAG-3	lymphocyte activation gene 3
MDSCs	myeloid-derived suppressor cells
MICA/B	major histocompatibility complex class I-related protein A/B
MMPs	matrix metalloproteinases
mTOR	mechanistic target of rapamycin
MUC1	mucin-1
NK	natural killer
NKp30, 44, 46	natural cytotoxicity receptors
NKG2A	natural killer group 2 member A
NKG2D	natural killer group 2 member D
NSCLC	non-small cell lung cancer
PD-1	programmed cell death protein 1
PD-L1	programmed death ligand 1
PGC-1α	peroxisome proliferator-activated receptor gamma coactivator 1-alpha
PGE2	prostaglandin E2
PSMA	prostate-specific membrane antigen
ROBO1	roundabout homolog 1
ROR1	receptor tyrosine kinase-like orphan receptor 1
ROS	reactive oxygen species
RNA	ribonucleic acid
scFvs	single-chain variable fragments
SIRPα/γ	signal regulatory protein-alpha/gamma
SLC1A5/SLC3A2/SLC7A5	amino acid transporters
STING	stimulator of interferon genes
TAAs	tumor-associated antigens
TAMs	tumor-associated macrophages
TGF	transforming growth factor
TGFβR	TGF-β receptor
TIGIT	T cell immunoreceptor with Ig and ITIM domains
TIM-3	T cell immunoglobulin and mucin domain containing-3
TM	transmembrane domain
TME	tumor microenvironment
TriKEs	trispecific killer cell engagers
TROP2	trophoblast cell surface antigen-2
